# Apoptotic cell death, detected *ex vivo* in peripheral blood lymphocytes of HIV-1 infected persons

**DOI:** 10.1155/S0962935196000543

**Published:** 1996-10

**Authors:** L. F. te Velde, I. Vermes, C. Haanen, C. P. M. Reutelingsperger, C. H. H. ten Napel

**Affiliations:** 1 Department of Internal Medicine, Oncology and AIDS section Medical Spectrum Twente Hospital Group Enschede The Netherlands; 2 Department of Clinical Chemistry Medical Spectrum Twente Hospital Group Enschede The Netherlands; 3 Department of Biochemistry University of Limburg Maastricht The Netherlands

## Abstract

In HIV-1 infection the ongoing depletion of CD4+ T-lymphocytes is believed, to a large extent, to be due to apoptosis. Until now quantitative information about *in vivo* apoptosis of lymphocytes in HIV-patients is scarce because of the very nature of the apoptotic process. Successful detection of apoptosis *ex vivo* requires the recognition of the initial phase of this process, because at a later stage the cells may not remain any longer in the circulation. We measured quantitatively the amount of early apoptotic peripheral blood lymphocytes directly *ex vivo* in HIV-1 infected patients using a recently described flow cytometric assay. With this method we observed in an unselected heterogenous group of twelve HIV-infected individuals a median percentage of apoptotic lymphocytes to be significantly higher than in ten healthy controls. To the best of our knowledge this is the first report of *ex vivo* observed increased apoptosis of peripheral blood lymphocytes in HIV-infected persons.


Mediators of Inflammation, 5, 379–381 (1996)

### Apoptotic cell death, detected *ex vivo* in peripheral blood lymphocytes of HIV-1 infected persons

L. F. te Velde,[1] I. Vermes,[2,CA] C. Haanen,[2]
C. P. M. Reutelingsperger[3] and C. H. H. ten Napel[1]

Departments of [1]Internal Medicine, Oncology and AIDS section, and [2]Clinical Chemistry, Medical Spectrum Twente, Hospital Group, Enschede; and [3]Department of Biochemistry, University of Limburg, Maastricht, The Netherlands

[CA]Corresponding Author
Fax: (+31) 53 487 3075
Email: i.vermes@pi.net

**In HIV-1 infection the ongoing depletion of CD4+ T-lymphocytes is believed, to a large extent, to be due to apoptosis. Until now quantitative information about *in vivo* apoptosis of lymphocytes in HIV-patients is scarce because of the very nature of the apoptotic process. Successful detection of apoptosis *ex vivo* requires the recognition of the initial phase of this process, because at a later stage the cells may not remain any longer in the circulation. We measured quantitatively the amount of early apoptotic peripheral blood lymphocytes directly *ex vivo* in HIV-1 infected patients using a recently described flow cytometric assay. With this method we observed in an unselected heterogenous group of twelve HIV-infected individuals a median percentage of apoptotic lymphocytes to be significantly higher than in ten healthy controls. To the best of our knowledge this is the first report of *ex vivo* observed increased apoptosis of peripheral blood lymphocytes in HIV-infected persons.**

**Key words:** Annexin V, Apoptosis, Flow cytometry, HIV infection

### Introduction

HIV-1 infection leads to a gradual depletion of CD4+ T-lymphocytes. This phenomenon cannot be explained solely by a direct cytopathic effect of HIV since the frequency of HIV infection is as minor as 1 in 100 000 cells in early infection.[1,2] Evidence is accumulating that the CD4+ cell loss is due to apoptosis to a large extent and that apoptosis of peripheral blood lymphocytes is a general feature in HIV-infected persons.[3] This important phenomenon is not limited to the CD4+ population because in advanced HIV-1 infection both CD4+ and CD8+ cells are primed for the cell death programme, by overexpression of the Fas antigen, a physiological receptor for apoptosis signalling.[4] Calculations of the turnover rate of peripheral CD4+ lymphocytes in HIV-1 infected patients resulted in estimates of $3 \times 10^7$ cells per day suggesting a high apoptotic rate.[5] These data could not been substantiated by direct measurements because, until now, assays that provide quantitative information about apoptosis lack specificity or sensitivity, are time consuming and usually require the destruction of cell integrity.

### Subjects and Methods

We measured quantitatively apoptosis of peripheral blood lymphocytes *ex vivo* in twelve HIV-1 infected individuals and in ten healthy controls using the APOPTEST™-FITC protocol (NeXins Research, the Netherlands), which numerates early apoptotic cells by probing for cell surface exposed phosphatidylserine (PS) with FITC labelled annexin V and for plasma membrane integrity by propidium iodide (PI) exclusion. This test discriminates between intact cells (FITC−/PI−), early apoptotic (FITC+/PI−) and necrotic cells (FITC+/PI+) as described elsewhere.[6,7] Procedures followed were in accordance with the policies of the Institutional Ethical Review Board of the Hospital Group.

### Results

The results of twelve consecutively measured peripheral blood samples of patients are given in Table 1. The amount of early apoptotic cells is expressed as percentage and absolute count of FITC-positive and PI-negative lymphocytes being flow cytometric recognized by their

© 1996 Rapid Science Publishers

**Table 1.** Amount of apoptotic peripheral blood lymphocytes *ex vivo* in an unselected group of 12 HIV infected individuals

| Patient | Lymphocytes (cells/mm$^3$) | Apoptotic cells (cells/mm$^3$) | (%) | CD4+ cells (cells/mm$^3$) | CD8+ cells (cells/mm$^3$) |
|---|---|---|---|---|---|
| 1 | 1 500 | 48 | (3.2) | 300 | 900 |
| 2 | 1 400 | 48 | (3.4) | 300 | 1 000 |
| 3 | 1 600 | 88 | (5.5) | 300 | 800 |
| 4 | 1 600 | 203 | (12.7) | 300 | 900 |
| 5 | 3 100 | 394 | (12.7) | 500 | 1 900 |
| 6 | 1 200 | 160 | (13.3) | 180 | 1 000 |
| 7 | 400 | 64 | (15.9) | 10 | 200 |
| 8 | 800 | 138 | (17.2) | 20 | 500 |
| 9 | 1 700 | 401 | (23.6) | 20 | 400 |
| 10 | nd | 113 | (28.2) | 10 | 600 |
| 11 | 700 | 221 | (31.5) | 190 | 400 |
| 12 | 700 | 42 | (34.5) | 50 | 400 |
| Mean | 1 336 | 177 | (16.8) | 182 | 750 |

nd = not done.

scatter properties. Absolute lymphocyte counts and CD4+ and CD8+ lymphocyte counts were measured separately. In healthy controls ($n$ = 10) a median of 2.1% (range 0.1–3.2) apoptotic lymphocytes were found whereas in HIV-infected patients the median was 16.8% (3.2–34.5; $p < 0.01$ Wilcoxon's two-sided rank-sum test). Eleven out of twelve patients had a CD4+ count of less than 400 cells/mm$^3$ and all patients had CD8+ cell counts in the normal range (200–1 100/mm$^3$).

### Discussion

The pathophysiological significance of apoptosis in the development of AIDS is not known: its inhibition in CD4+ Jurkat T-cell lines enhances virus production and infection *in vitro*.[8] Reports about correlations between apoptosis and progression to disease show conflicting results.[9,10] Until now there is little quantitative data about the occurrence, duration and frequency of apoptosis *in vivo* in HIV infection because of the very nature of the process. Detection of the earliest phase of apoptosis *ex vivo* is of paramount importance for measuring apoptotic cells before they are destroyed by phagocytes.[11] Our observation shows for the first time the presence of a considerable degree of apoptosis in circulating lymphocytes of HIV infected persons. We observed in an unselected, heterogenous group of twelve HIV-1 infected individuals a median percentage of apoptotic lymphocytes to be significantly higher than in ten healthy controls. Patients nos 11 and 12 who showed the highest percentage of apoptotic lymphocytes were treated for ongoing major opportunistic infections (cerebral toxoplasmosis and pulmonary tuberculosis). Probably this infection served as an additional immunological stimulant. In HIV-infected patients this paradoxically induces apoptosis instead of proliferation as the result of an inappropriate re-emergence of the programmed cell death pathway as described after *in vitro* stimulation.[12] Our experience shows that the annexin V assay is a feasible and relatively simple method for *ex vivo* detection of early apoptotic lymphocytes. To the best of our knowledge this is the first report of *ex vivo* observed increased apoptosis of peripheral blood lymphocytes in HIV-infected persons.

### References

1. Schnittman SM, Psallidopoulos MC, Lane HC, *et al.* The reservoir for HIV-1 in human peripheral blood is a T-cell that maintains expression of CD4. *Science* 1989; **245**: 305–308.
2. Brinchmann JE, Albert J, Vartdal F. Few infected C4+ T cells but a high proportion of replication-competent provirus copies in asymptomatic human immunodeficiency virus type I infection. *J Virol* 1991; **65**: 2019–2023.
3. Ameisen JC. Programmed cell death (apoptosis) and cell survival regulation: relevance to AIDS and cancer. *AIDS* 1994; **8**: 1197–1213.
4. Silvestris F, Cafforio P, Frassanito MA, *et al.* Overexpression of Fas antigen on T cells in advanced HIV-1 infection: differential ligation constantly induces apoptosis. *AIDS* 1996; **10**: 131–141.
5. Ho DD, Neumann AU, Perelson AS, Chen W, *et al.* Rapid turnover of plasma virions and CD4 lymphocytes in HIV-1 infection. *Nature* 1995; **373**: 123–126.
6. Koopman G, Reutelingsperger CP, Kuijten GA, *et al.* Annexin V for flow cytometric detection of phosphatidylserine expression on B cells undergoing apoptosis. *Blood* 1994; **84**: 1415–1420.
7. Vermes I, Haanen C, Steffens-Nakken H, Reutelingsperger C. A novel assay for apoptosis, flow cytometric detection of phosphatidylserine expression on early apoptotic cells using fluorescein labelled annexin V. *J Immunol Meth* 1995; **184**: 39–51.
8. Antoni BA, Sabbatini P, Rabson AB, White E. Inhibition of apoptosis in human immunodeficiency virus-infected cells enhances virus production and facilitates persistent infection. *J Virol* 1995; **69**: 2384–2392.
9. Meyaard L, Otto SA, Keet IP, Roos MT, Miedema F. Programmed death of T cells in human immunodeficiency virus infection. No correlation with progression to disease. *J Clin Invest* 1994; **93**: 982–988.
10. Gougeon ML, Lecoeur H, Dulioust A, *et al.* Programmed cell death in peripheral lymphocytes from HIV-infected persons. Increased susceptibility to apoptosis of CD4 and CD8 T cells correlates with lymphocyte activation and with disease progression. *J Immunol* 1996; **156**: 3509–3520.
11. Carbonari M, Cibati M, Fiorilli M. Measurement of apoptotic cells in peripheral blood. *Cytometry* 1995; **22**: 161–167.
12. Groux H, Monte D, Bourrez JM, et al. Activation of CD4+ T-lymphocytes in asymptomatic HIV infected patients induces the programmed action of lymphocyte death by apoptosis. *C R Acad Sci III* 1991; **312**: 599–606.

**Received 1 August 1996; accepted 16 August 1996**
